# Complete Remission in Metastatic Pheochromocytoma Treated with Extensive Surgery

**DOI:** 10.7759/cureus.447

**Published:** 2016-01-05

**Authors:** Claudia Arnas-Leon, Víctor Sánchez, Ana D. Santana Suárez, Sara Quintana Arroyo, Carmen Acosta, Francisco Javier Martinez Martin

**Affiliations:** 1 Endocrinology and Nutrition, Hospital Universitario de Gran Canaria Doctor Negrín; 2 Radiology, Hospital Universitario de Gran Canaria Doctor Negrín; 3 Endocrinology, Clinica San Roque

**Keywords:** pheochromocytoma, metastatic pheochromocytoma, malignant pheochromocytoma, metanephrines, normetanephrines, extensive surgery, catecholamines

## Abstract

Pheochromocytomas are rare neuroendocrine tumors that arise from chromaffin cells of the adrenal medulla. Malignant pheochromocytoma is defined as the presence of metastatic spread in tissues where chromaffin cells are not usually present. This case report describes the case of a relapsed malignant pheochromocytoma, spread to the right liver lobe, superior pole of the right kidney, posterior right hemidiaphragm, right hemidiaphragmatic pillar, inferior vena cava, and regional lymph nodes. After evaluation, an extensive surgery was performed, with resection of all the affected tissues and regional lymphadenectomy. No adjuvant treatment (radiotherapy or chemotherapy) was given. Complete clinical, biochemical, and radiological remission was achieved, with normalisation of metanephrine and normetanephrine. To date, six years after surgery, the patient remains asymptomatic and normotensive without taking any antihypertensive medication. We conclude that the therapeutic approach should be individualized in the case of metastatic pheochromocytoma. Extensive surgery can be considered as a treatment option, even in the case of multiple metastases, as it may be able to achieve complete remission of the disease, avoiding costly and potentially dangerous adjuvant therapies.

## Introduction

Pheochromocytomas are rare catecholamine-secreting tumors of the adrenal medulla, with an estimated annual incidence of 8 per 100,000 person-years. The prevalence is increasing due to advanced imaging tests with a high rate of incidental adrenal mass [1].

Classic clinical presentation of pheochromocytoma consists of headache, sweating, flushing or pallor, and episodes of paroxysmal hypertension and tachycardia. Less frequent symptoms are orthostatic hypotension, hyperglycemia, and cardiomyopathy. The patient might be asymptomatic as well [2]. Thus, the main associated morbidity and mortality remains on the difficult management of hypertensive crisis. In the worst scenario, cardiovascular complications and eventually death may occur (myocardial infarction, heart failure, or stroke).

The diagnosis is typically confirmed by measurements of urinary and plasma fractionated metanephrines and catecholamines [3]. After the biochemical confirmation, a radiological evaluation should be performed to locate the tumor. Abdominal computer tomography (CT) scan and magnetic resonance imaging (MRI) are the standard imaging tests, but ^131^I-MIBG is particularly useful in confirming the functionality of the tumor and also in locating metastatic disease or extra-adrenal primary tumors (paragangliomas). Almost 95% of the tumors are inside the abdomen and pelvis [4]. 

Although there are different treatment options, laparoscopic resection is considered the first-line therapy option, because total resection ensures the best chance of complete remission on a potentially lethal disease [5]. It is necessary to spotlight the importance of adequate blocking of the alpha adrenergic receptors with intravenous or oral medication, before and during the surgery, to avoid perioperative complications. Other treatment options are radiotherapy for bone injury, ^131^I-MIBG for unresectable malignant pheochromocytomas, or chemotherapy with cyclophosphamide for metastatic disease [6].

According to the 2004 World Health Organization (WHO) criteria, a pheochromocytoma should be considered as malignant if metastatic spread exists [7]. Metastatic disease in pheochromocytoma is infrequent (5-26%) and may be present on the initial diagnosis or after surgical removal. Most treatments are palliative in these cases, and without treatment, the survival is less than 50% in five years [8].

## Case presentation

We present the case of a 58-year-old female patient with an unremarkable personal history of chronic diseases, except colonic and sigmoid diverticulosis. The patient denied any headache or palpitations and took no medications. The physical examination revealed mild hypertension (150/90 mmHg) and tachycardia (100 bpm) without any other pathological findings. Informed patient consent was obtained.

During a hospital admission for acute diverticulitis, an abdominal CT scan was performed. An incidental solid adrenal mass of 8 x 5.5 x 9 cm was found on the right adrenal gland with extension to the right lobe of the liver and compression of the inferior vena cava and collateral perirenal circulation. A right adrenalectomy was carried out without postsurgical complications, with an anatomopathological final diagnosis of pheochromocytoma.

**Figure 1:**
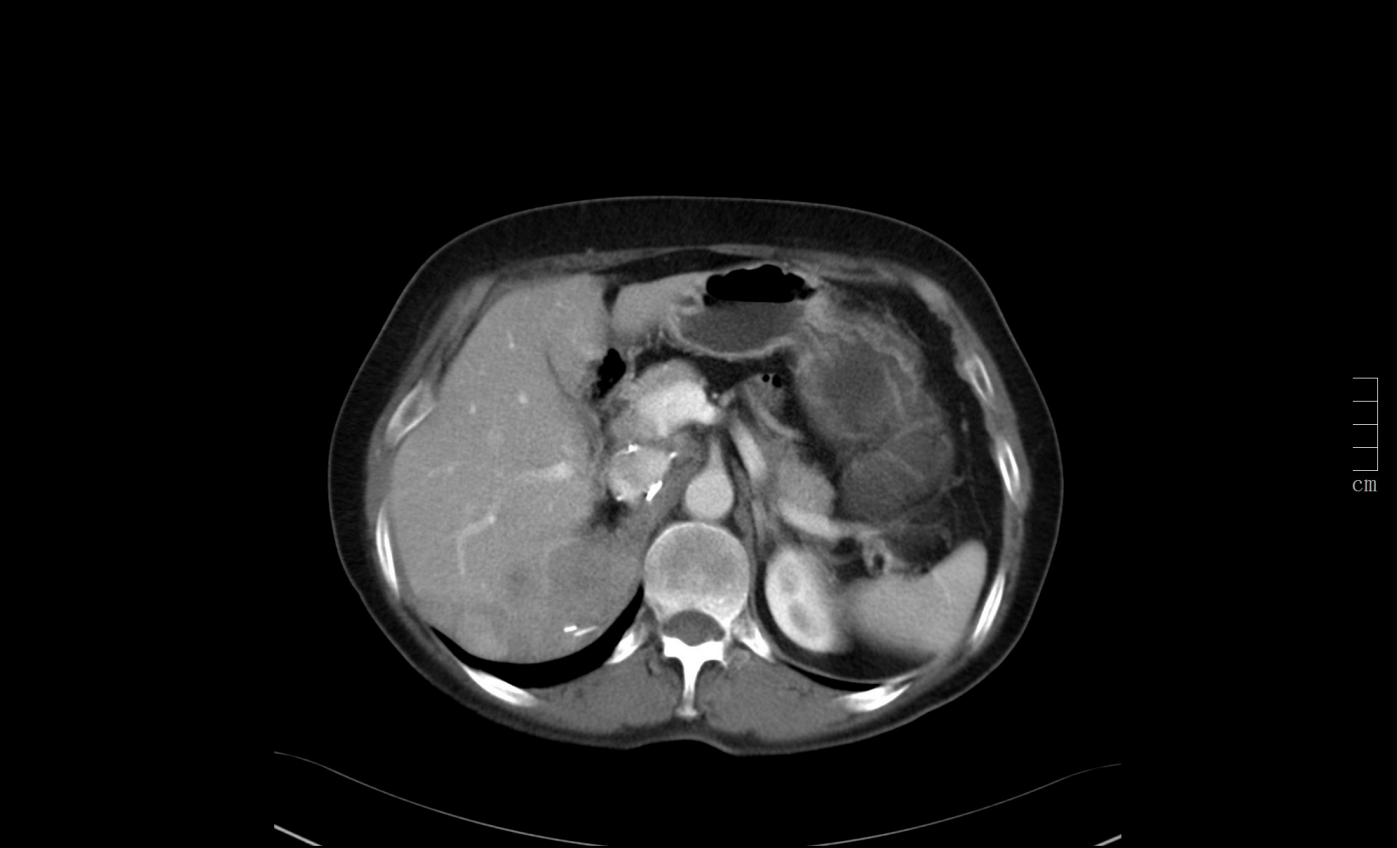
Metastatic spread of pheochromocytoma in abdominal CT

**Figure 2:**
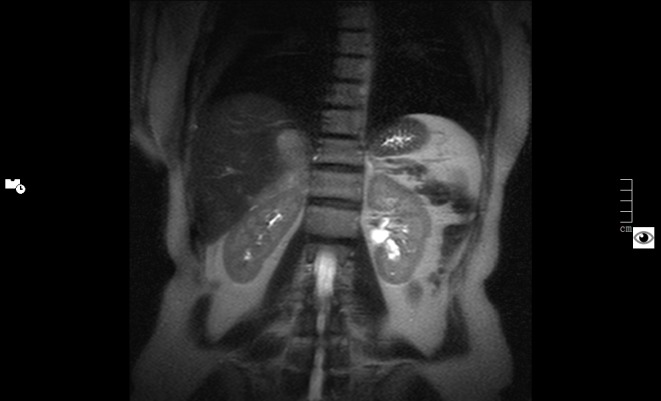
Metastatic spread of pheochromocytoma in right liver lobe, right kidney, and right hemidiaphragm in abdominal MRI

Twelve years later, a control abdominal CT scan showed a 9 x 4.5 cm irregular and heterogeneous mass in the surgical area with poorly defined outlines, which had spread to the right lobe of the liver, the superior pole of the right kidney, the posterior right hemidiaphragm and right hemidiaphragmatic pillar, and the inferior vena cava, demonstrating metastatic recurrence of a malignant pheochromocytoma (as seen in the abdominal CT in Figure 1 and abdominal MRI in Figure 2).

The patient denied abdominal pain and remained asymptomatic. At this point, the patient was referred to our Hypertension Clinic.

Lab tests showed routine blood chemistries and CBC without any relevant findings, except for increased catecholamines: serum metanephrine > 1.200 pg/ml (0-90), serum normetanephrine >1.600 pg/ml (0-180), urine metanephrine 23.264 ug/24h (52-341), urine normetanephrine 18.915 ug/24h (88-444), serum adrenalin 302 pg/ml (20-60), serum noradrenalin 995 pg/ml (135-650), and serum dopamine 33 pg/ml (10-150).

**Figure 3:**
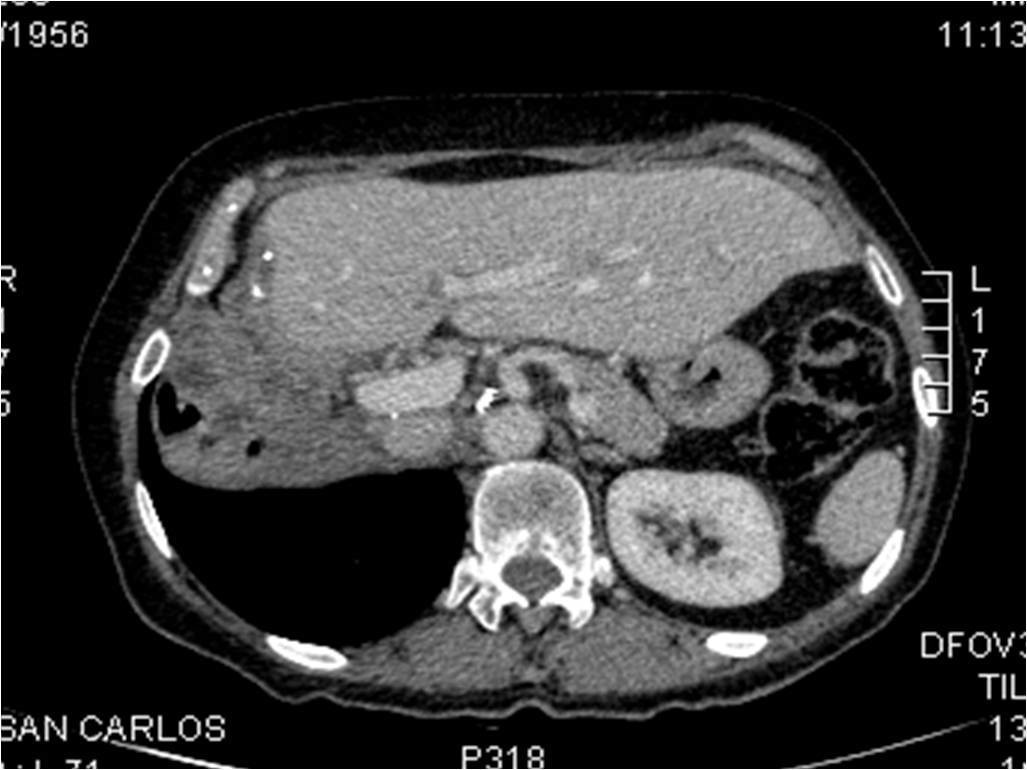
Abdominal CT scan after extensive surgery

The patient was admitted, and the case was discussed in a multidisciplinary expert committee. An extensive surgery was performed, with a right lobar hepatectomy, right nephrectomy, right hemidiaphragm partial resection, perivascular, and retroperitoneal and right liver lobe lymphadenectomy (as can be seen in the abdominal CT after surgery in Figure 3).

The intra-surgical biopsy confirmed the diagnosis of metastatic pheochromocytoma. There were no complications during surgery nor in the early and late postsurgical periods. The relapsed pheochromocytoma was entirely resected, and lab tests showed normalisation of the catecholamines. Additional treatment was therefore not required after the extensive surgery.

To date, the patient has been evaluated annually for six years and remains asymptomatic and normotensive without taking any antihypertensive medication. Annual abdominal CT scans and lab tests have revealed no signs of pheochromocytoma complications with metanephrine and normetanephrine levels consistently remaining in the low-normal range (Table 1).

**Table 1:** Metanephrine and normetanephrine follow-up

	09/09	09/10	05/11	11/12	06/13	05/14	06/15
Metanephrine (serum)	> 1200 pg/ml	30 pg/ml	72 pg/ml	34 pg/ml	35 pg/ml	70 pg/ml	148 pg/ml
Normetanephrine (serum)	> 1600 pg/ml	94 pg/ml	141 pg/ml	113 pg/ml	47 pg/ml	119 pg/ml	137 pg/ml

## Discussion

Pheochromocytomas are neuroendocrine tumors that arise from chromaffin cells of the adrenal medulla. Only 5-26% are malignant according to large series, and malignancy requires evidence of metastases and tumor spread in sites where chromaffin tissue is not normally present. Neither the radiologic appearance of the tumor nor its pathology allow for the diagnosis of malignancy [8].

Imaging tests are necessary in order to document tumor spread. The most recommended imaging studies are CT and MRI scans, with a sensitivity of 77-98% and 90-100%, respectively, and a specificity of 29-92% and 50-100%, respectively, in the localization of adrenal or extra-adrenal tumors [9].

Pheochromocytomas usually enhance avidly on CT scans, and even when they may wash out like adrenal adenomas, these tumors tend to enhance greater in an arterial or portal venous contrast phase. One hundred and ten Hounsfield Units (HU) of enhancement on the arterial phase indicates pheochromocytoma, and metastases typically are hypervascular and radiologically hyperintense. According to scientific literature, the use of contrast may cause hypertensive crisis so it should be used carefully [10].

The most significant property of these tumors on MRI scans is that they are usually shown as hyperintense on T2, which practically confirms a pheochromocytoma [10].

However, metastases or local recurrences may be microscopic at diagnosis and become apparent some years later. This is why it is recommended to perform a yearly follow-up with lab tests that include metanephrines and normetanephrines [8].

Surgery is considered the first line therapeutic option, and it should be considered even in the case of tumor spread. Resection of distant metastases decreases catecholamine secretion and, therefore, reduces the cardiovascular risk factors and, ultimately, the risk of death from cardiovascular complications [8]. All of this contributes to relieve the patient’s symptoms, leading to an improved quality of life [9]. 

In the case of malignant disease, surgery, as the only option of treatment, is rarely curative. The five-year survival rate of these patients varies between 34% and 60%, mainly depending on the location of the metastatic lesions [9].

Alternatives to surgical resection consist of radiometabolic treatment with ^131^I-MIBG, radiofrequency ablation, external radiotherapy, and use of antineoplastic agents, such as cyclophosphamide, vincristine, and dacarbazine [8]. Unfortunately, none of these therapies usually increase the survival on their own and a combination of them is often needed to get a satisfactory result. However, the initial approach with any of these therapy options, especially in the case of chemotherapy, may be useful in order to reduce the tumor size before surgery [9].

## Conclusions

In conclusion, metastatic pheochromocytoma is a rare and challenging disease, and there are several options for treatment, from chemotherapy to surgery. We have presented the case of a female patient affected by an infrequently large malignant pheochromocytoma spread to vital organs with an extraordinary response to extensive surgery who did not require any other additional treatment.

We recommend that an individual approach is needed and extensive surgery should be considered in patients who may benefit from this type of therapy in order to avoid chronic medications with undesirable side effects.
